# Dataset on political connections, Sharia, and abnormal returns surrounding M&A announcement in the Indonesian stock market

**DOI:** 10.1016/j.dib.2021.107378

**Published:** 2021-09-20

**Authors:** Budi Wahyono

**Affiliations:** a The Graduate School of East Asian Studies, Yamaguchi University, Yamaguchi 753-8514, Japan; b Department of Economics Education, Faculty of Teacher Training and Education, Universitas Sebelas Maret, Surakarta 57126, Indonesia

**Keywords:** Political connections, Sharia, Abnormal returns, M&A, Indonesia

## Abstract

This article presents a dataset on political connections, Sharia, and abnormal returns surrounding the M&A announcement of listed firms on Indonesia Stock Exchange (IDX) during the period 2010–2016. The dataset provides both short-run and long-run abnormal returns. Using an event study methodology, I calculate cumulative abnormal returns (CAR) as short-run abnormal returns and buy-and-hold abnormal returns (BHAR) as long-run abnormal returns. This dataset may be useful for researchers who study political connections, Sharia, and M&A performance. The data presented in this article are related to the research article entitled “Political connections, Sharia and M&A performance: Evidence from Indonesia” (Wahyono, 2021) [1].

**Specifications Table**

**Table:** 

Subject	Finance and Banking
Specific subject area	Corporate finance
Type of data	XLSX file and tables within the article
How data were acquired	Data on M&A deals between 2010 and 2016 were obtained from the Commission for Supervision of Business Competition (KPPU), while the dates of M&A announcements were collected from firms’ official websites, annual reports, and online newspapers. I sourced daily stock prices from Yahoo Finance, then used Excel to calculate CAR and BHAR. I manually traced political connections data from firms’ annual reports. Data on Sharia shares were taken from IDX and Financial Services Authority (OJK). Finally, other related firms’ financial data were collected from financial reports and annual reports.
Data format	Raw Analyzed
Description of data collection	The data provided the short-run abnormal returns (CAR) and long-run abnormal returns (BHAR) surrounding M&A announcements. I then calculated CAR and BHAR for politically connected firms, Sharia-compliant firms, politically connected Sharia-compliant firms, and non-politically connected non-Sharia-compliant firms.
Data source location	The data sources are presented in Table 1.
Data accessibility	The data can be found on Mendeley Data: http://dx.doi.org/10.17632/xr3ny45x2g.1
Related research article	B. Wahyono, Political connections, Sharia and M&A performance: Evidence from Indonesia, J. East Asian Stud. 19 (2021) 105–120 [1]

**Value of the Data**

• The dataset provides short-run and long-run abnormal returns of firms that carried out M&A in the Indonesian market between 2010 and 2016, especially for firms with political connections and Sharia shares. I determine the date of the M&A announcement as the date on which the deal was publicly announced. This dataset is useful for investigating the short-run and long-run performance of M&A in Indonesia.
• The dataset contains firm-level data such as political connections, Sharia shares, abnormal returns, and various financial data (firm size, leverage, ROA, risk, and growth). Consequently, the dataset is particularly useful for those studying M&A performance (both short-run and long-run) and the role of political connections and Sharia compliance.
• The dataset can also be compared with other datasets that apply different Sharia indexes, political connections' criteria, and measurements of abnormal returns to study similar phenomena.

## 1 Data Description

The unbalanced panel comprises 48 M&A deals from 40 non-financial public firms in Indonesia covering the period 2010 to 2016.^1^ I chose 2010 as the start of the study period because KPPU published its first list of M&A deals that year. The study period ended in 2016 because I chose to calculate long-run abnormal returns two years and three years after each M&A announcement. The dataset contains firm-level data on political connections, Sharia shares, cumulative abnormal returns (CAR), buy-and-hold abnormal returns (BHAR), and various financial measures (firm size, leverage, return on assets [ROA], risk, and growth).

**Fig. 1:**
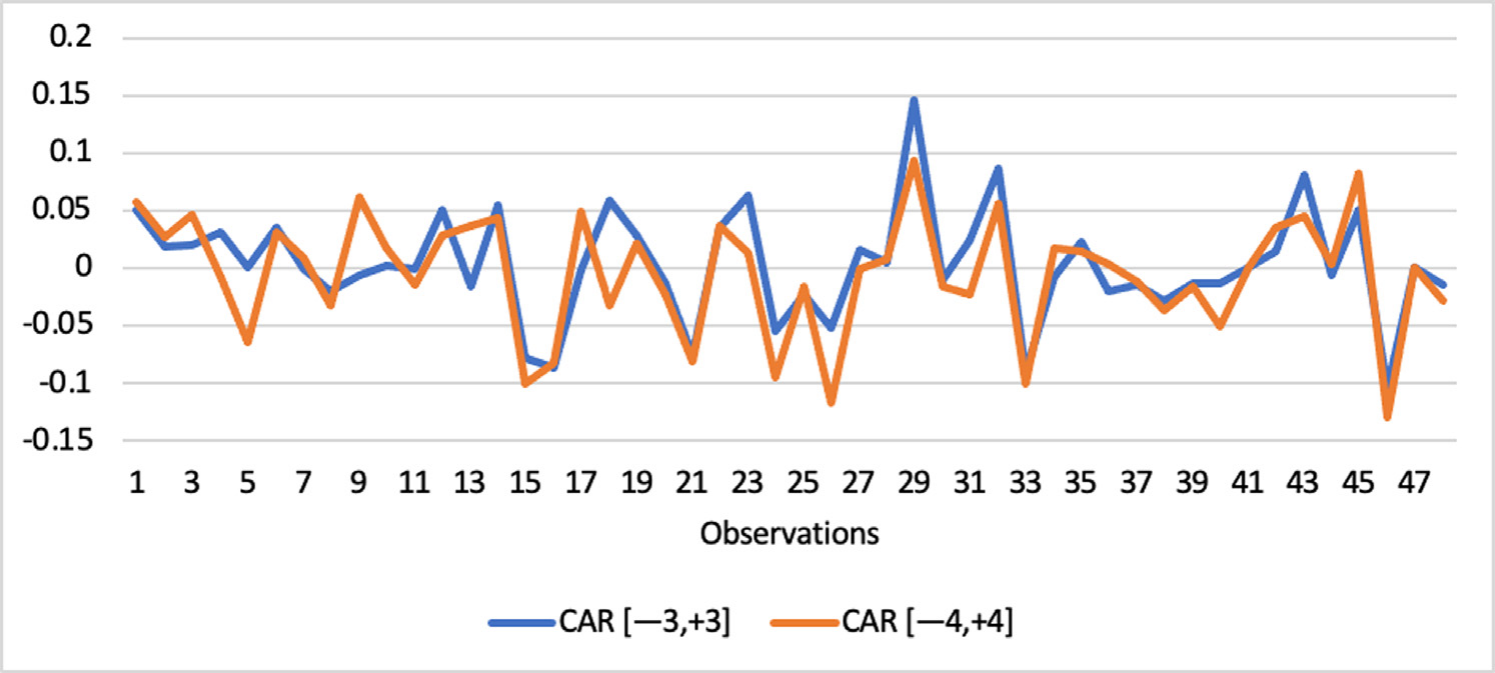
CAR of full sample.

**Fig. 2:**
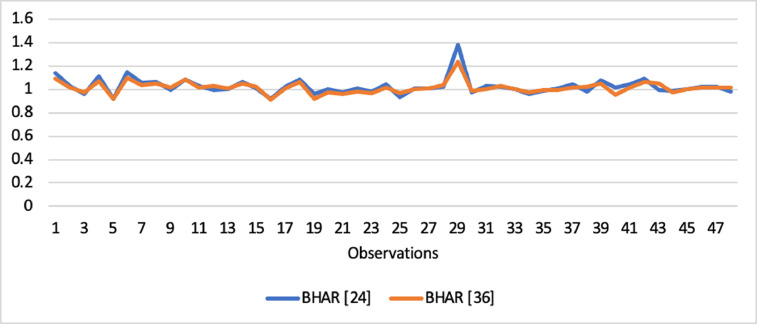
BHAR of full sample.

**Fig. 3:**
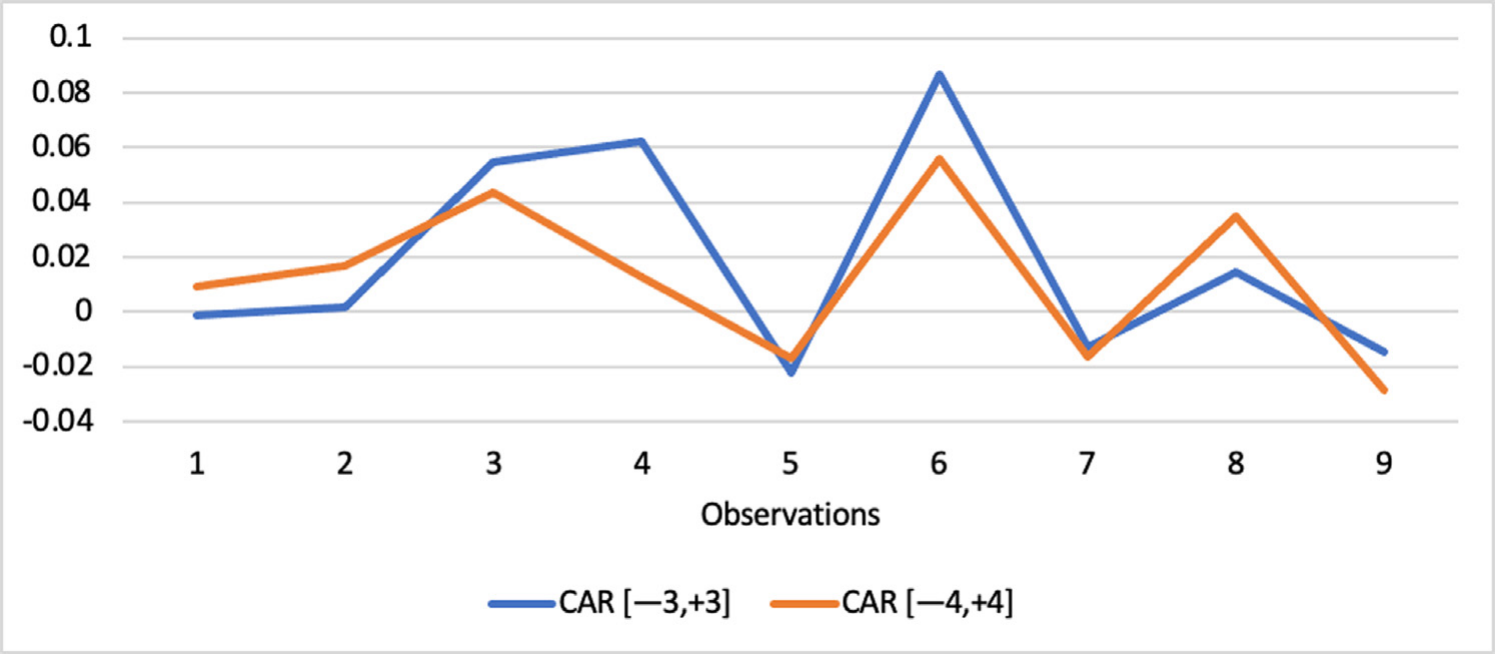
CAR of politically connected firms.

**Fig. 4:**
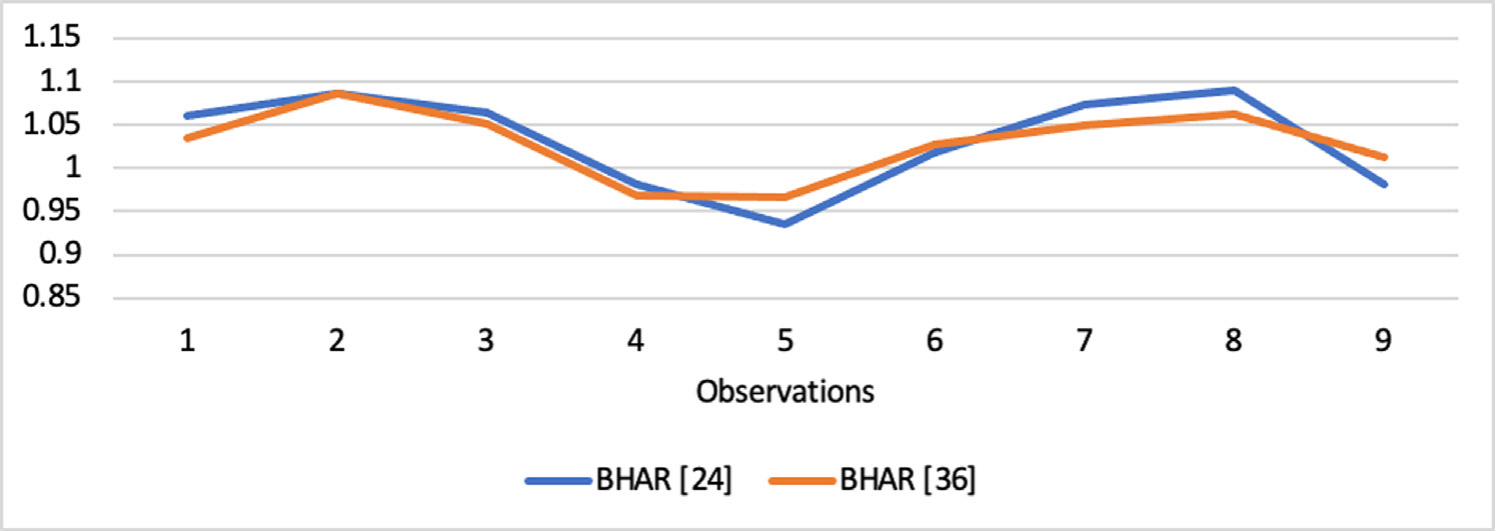
BHAR of politically connected firms.

**Fig. 5:**
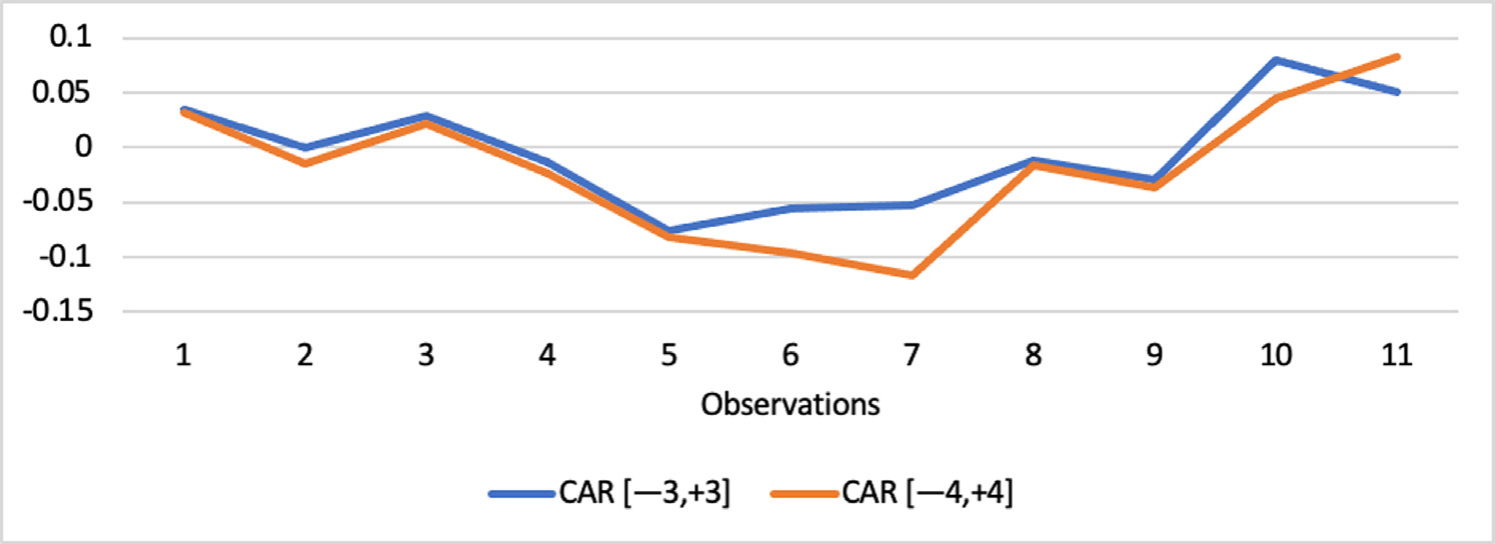
CAR of Sharia-compliant firms.

**Fig. 6:**
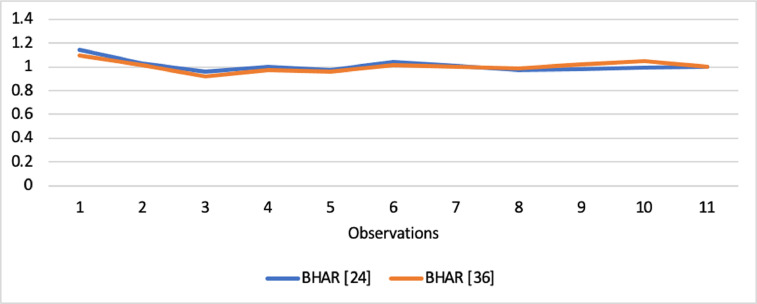
BHAR of Sharia-compliant firms.

**Fig. 7:**
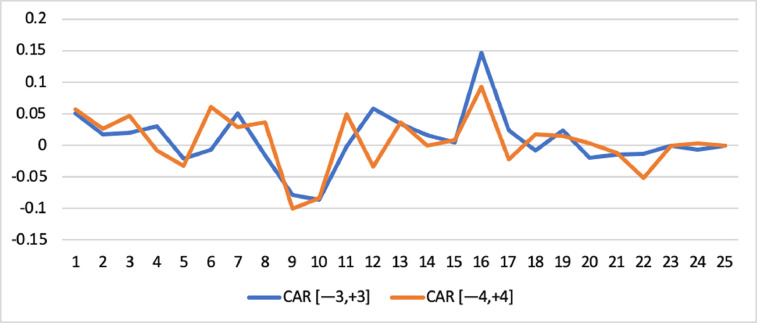
CAR of politically connected Sharia-compliant firms.

**Fig. 8:**
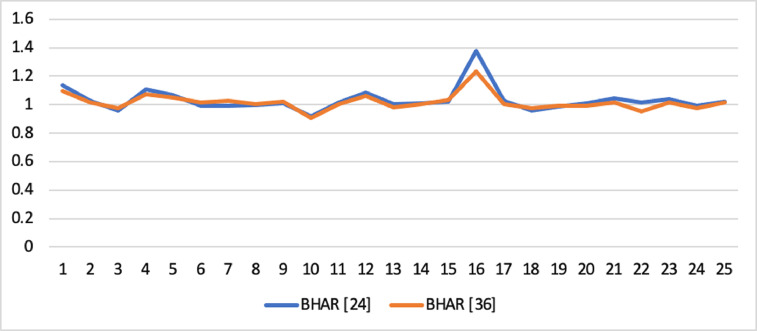
BHAR of politically connected Sharia-compliant firms.

**Fig. 9:**
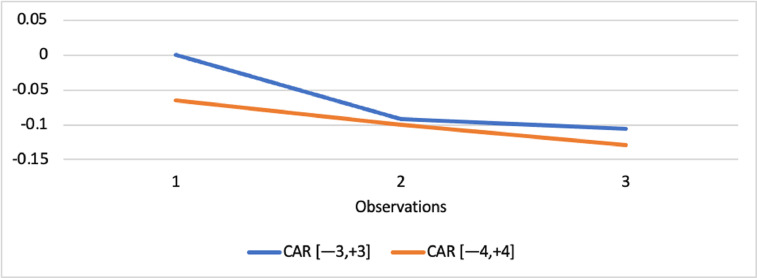
CAR of non-politically connected non-Sharia-compliant firms.

**Fig. 10:**
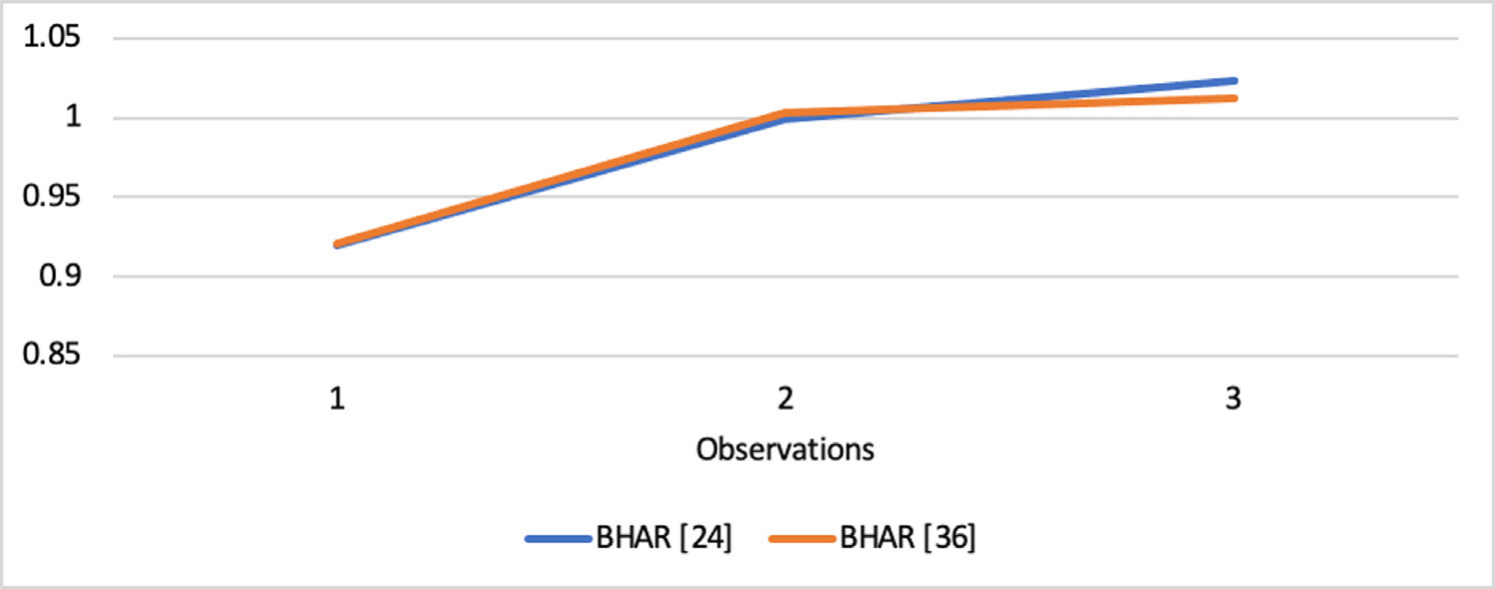
BHAR of non-politically connected non-Sharia-compliant firms.

Short-run abnormal returns–measured by CAR (long-run abnormal returns–measured by BHAR) for the full sample are presented in Figs. 1, 2. Fig. 3 shows CAR for politically connected firms, and Fig. 4 shows BHAR for politically connected firms. In addition, CAR (BHAR) for Sharia-compliant firms is displayed in Figs. 5, 6. Fig. 7 presents CAR for politically connected Sharia-compliant firms, while Fig. 8 presents BHAR for politically connected Sharia-compliant firms. Finally, CAR for non-politically connected non-Sharia-compliant firms is displayed in Fig. 9, while their BHAR is displayed in Fig. 10.

## 2 Experimental Design, Materials and Methods

As mentioned in the previous section, my dataset contains political connections, Sharia compliance, CAR, BHAR, and various financial data (firm size, leverage, ROA, risk, and growth). Table 1 presents the summary of variable definitions and data sources for each variable.

**Table 1:** Variable definitions and their data sources.

Variable	Definition	Data source
Political connections	A dummy variable, equal to 1 if politically connected firm, and 0 otherwise	Firms’ annual reports obtained from IDX (https://idx.co.id/) and firms’ official websites
Sharia	A dummy variable, equal to 1 if firm has Sharia shares, and 0 otherwise	IDX (https://idx.co.id/) and OJK (https://www.ojk.go.id/)
CAR [–3, +3]	Cumulative abnormal returns with event window three days before to three days after the M&A announcement	To calculate CAR, I use stock prices and composite stock index data taken from Yahoo Finance (https://finance.yahoo.com/)
CAR [–4, +4]	Cumulative abnormal returns with event window four days before to four days after the M&A announcement	To calculate CAR, I use stock prices and composite stock index data taken from Yahoo Finance (https://finance.yahoo.com/)
BHAR [24]	Buy-and-hold abnormal returns with event window twenty-four months after M&A announcement	To calculate BHAR, I use stock prices and composite stock index data taken from Yahoo Finance (https://finance.yahoo.com/)
BHAR [36]	Buy-and-hold abnormal returns with event window thirty-six months after M&A announcement	To calculate BHAR, I use stock prices and composite stock index data taken from Yahoo Finance (https://finance.yahoo.com/)
Firm size	The natural logarithm of total assets	Firms’ financial reports and annual reports obtained from IDX (https://idx.co.id/) and firms’ official websites
Leverage	The sum of total short-term and total long-term debt divided by total assets	Firms’ financial reports and annual reports obtained from IDX (https://idx.co.id/) and firms’ official websites
ROA	Return on assets–measured by the ratio of net income divided by total assets	Firms’ financial reports and annual reports obtained from IDX (https://idx.co.id/) and firms’ official websites
Risk	The daily stock return standard deviation [—60, —1] before the M&A announcement	To calculate risk, I use stock price data from Yahoo Finance (https://finance.yahoo.com/)
Growth	The growth rate of total assets over the last fiscal year	Firms’ financial reports and annual reports obtained from IDX (https://idx.co.id/) and firms’ official websites

### 2.1 Political connections

Following Faccio [2] and Habib et al. [3], I treat a firm as politically connected if it is state-owned or if the larger shareholders (>10% ownership) or the top management are currently or were formerly (a) members of parliament, (b) ministers or heads of local government, or (c) closely connected with top officials.

### 2.2 Sharia

A firm is considered as Sharia compliant if it has Sharia shares, i.e., is a constituent of the Indonesia Sharia Stock Index (ISSI).^2^

### 2.3 Abnormal returns

I rely on an event study methodology to measure both short-run and long-run abnormal returns surrounding M&A announcement.

#### 2.3.1 Cumulative abnormal returns (CAR)

First, I employ a basic market model to estimate the alpha and beta of the sample firm:

$$R_{it}=\alpha_{i}+\beta_{i}R_{mt}+\varepsilon_{it},$$

where *R_it_* is *s* the daily return for firm *I* on day *t*, and *R_mt_* is the daily return for market index *m* on day *t*. The estimation window is defined from 180 days to 30 days (150 trading days in total) prior to the M&A announcement. Then, using the basic market model, the abnormal return of security *i* for period *t* is:

$$A_{i,t}=R_{i,t}-\alpha_{i}-\beta_{i}R_{m,t},$$

where α and β are estimated market model coefficients. Finally, I calculate the CAR using the following specification:

$$CAR\left[t_{1},t_{2}\right]=\sum_{t=t_{1}}^{t_{2}}A_{i,t}.$$

I use three days before to three days after the M&A announcement (CAR [—3, +3]) and four days before to four days after the M&A announcement (CAR [—4, +4]) as the event window.

#### 2.3.2 Buy-and-hold abnormal returns (BHAR)

Following Barber & Lyon [4], I calculate *BHAR* as follows:

$$BHAR_{i,t}=\prod_{t=0}^{t}\left(1+R_{i,t}\right)-\prod_{t=0}^{t}\left(1+R_{benchmark,t}\right),$$

where *R_i,t_* is the realized return of security *i* on day *t*. I use market return on day *t*, which I denote as *R_benchmark_*. The mean market-adjusted BHAR is defined as:

$$\bar{BHAR}=\;\frac{1}{n}\sum_{t=0}^{t}BHAR_{i,t}.$$

I use twenty-four months after the M&A announcement (BHAR [24]) and thirty-six months after the M&A announcement (BHAR [36]) as the event window.

### 2.4 Other financial data

This dataset also includes firm-level financial data on size, leverage, ROA, risk, and growth. Firm size is calculated as the natural logarithm of total assets. I calculate leverage by the sum of total short-term and total long-term debt divided by total assets. ROA is measured as the ratio of net income divided by total assets. I measure risk by the daily stock return standard deviation [—60, —1] before the M&A announcement. Finally, growth is calculated as the growth rate of total assets over the last fiscal year.

## Ethics Statement

The author declares that this work does not involve the use of human subjects.

## CRediT authorship contribution statement

**Budi Wahyono:** Conceptualization, Formal analysis, Investigation, Data curation, Writing – original draft, Writing – review & editing.

## Declaration of Competing Interest

The author declares that he has no known competing financial interests or personal relationships that could have appeared to influence the work reported in this paper.

## Footnotes

1 Although there are 322 M&A deals over the 2010–2016 period, I restrict my sample to only non-financial listed firms in the IDX, firms that have not conducted M&A more than once within three months, and firms without missing data.

2 Because ISSI was published on May 12, 2011, I also use data from the Sharia Securities List (DES) and the Jakarta Islamic Index (JII) for firms that announced M&A before that date. DES is a collection of Sharia Securities in the capital market launched by OJK. JII is the first Sharia stock index in the Indonesian capital market, that consists of only the 30 most liquid Sharia stocks listed in the IDX.
